# Positive Effects of Guanidinium Salt Post-Treatment on Multi-Cation Mixed Halide Perovskite Solar Cells

**DOI:** 10.3390/nano14131161

**Published:** 2024-07-07

**Authors:** Damir Aidarkhanov, Ikenna Henry Idu, Xianfang Zhou, Dawei Duan, Fei Wang, Hanlin Hu, Annie Ng

**Affiliations:** 1 Department of Electrical and Computer Engineering, Nazarbayev University, Astana 010000, Kazakhstan; aidarkhanov@nu.edu.kz (D.A.); ikennahenry.idu@nu.edu.kz (I.H.I.); duan.dawei@nu.edu.kz (D.D.); 2 Hoffmann Institute of Advanced Materials, Shenzhen Polytechnic University, 7098 Liuxian Boulevard, Shenzhen 518055, China; 257508@whut.edu.cn (X.Z.); 321087@whut.edu.cn (F.W.); 3 National Laboratory Astana, Nazarbayev University, Astana 010000, Kazakhstan

**Keywords:** perovskites, solar cells, interface engineering, dimensional engineering

## Abstract

As one of the most promising photovoltaic technologies, perovskite solar cells (PSCs) exhibit high absorption coefficients, tunable bandgaps, large carrier mobilities, and versatile fabrication techniques. Nevertheless, the commercialization of the technology is hindered by poor material stability, short device lifetimes and the scalability of fabrication techniques. To address these technological drawbacks, various strategies have been explored, with one particularly promising approach involving the formation of a low-dimensional layer on the surface of the three-dimensional perovskite film. In this work, we demonstrate the use of guanidinium tetrafluoroborate, CH_6_BF_4_N_3_, (GATFB) as a post-treatment step to enhance the performance of PSCs. Compared with the control sample, the application of GATFB improves the film surface topology, reduces surface defects, suppresses non-radiative recombination, and optimizes band alignment within the device. These positive effects reduce recombination losses and enhance charge transport in the device, resulting in PSCs with an open-circuit voltage (*V_OC_*) of 1.18 V and a power conversion efficiency (PCE) of 19.7%. The results obtained in this work exhibit the potential of integrating low-dimensional structures in PSCs as an effective approach to enhance the overall device performance, providing useful information for further advancement in this rapidly evolving field of photovoltaic technology.

## 1. Introduction

Hybrid organic–inorganic perovskite solar cells (PSCs) are one of the most promising photovoltaics among all emerging solar cell technologies and demonstrate impressive power conversion efficiencies (PCEs) for single junction cells with the latest value reaching 26.54% [1]. The current record PCEs were reached in a relatively short time since the first application of organometallic halide perovskites in photovoltaics by Kojima et al. [2] in 2009. Such remarkable progress is attributed to the extraordinary optical and electronic properties of perovskite materials [3]. Despite the significant progress in PSCs, the commercialization of perovskite technology still needs to overcome challenges, including lifetime issues due to the instability of PSCs towards moisture and oxygen [4,5,6].

Many passivation strategies were proposed, such as dimension engineering, additive engineering, and interface optimization, which can reduce the sensitivity of devices to humidity and oxygen and enhance the overall stability of PSCs [7,8,9,10]. In the bulk 3D perovskite layer BX_6_^4−^, octahedra are connected to others in all three dimensions. In comparison, the octahedra of 2D perovskites are only connected in two dimensions forming slabs and in the third dimension they are separated by organic molecules. Such structural differences enhance hydrophobic properties and stability and suppress ion migration in 2D perovskites [11,12,13]. Apart from improving stability, the dimensionality reduction due to quantum confinement effects increases band gap values and creates blocking layers for the charge carriers [14]. Therefore, 2D perovskite absorbers demonstrate reduced light absorption and hindered charge transport. To enhance the stability of perovskite devices without compromising charge extraction efficiency, one approach is to form a 2D capping layer on the surface of a 3D perovskite film. This is commonly achieved by the post-treatment of the perovskite layer using a solution of large organic cation salts.

The most common organic cations used in dimensional engineering are n-butylammonium (BA^+^) [15,16], phenetylammonium (PEA^+^) [17,18,19], and 5-ammoniumvaleric acid (5-AVA^+^) [20,21]. G. Li et al. [16] devised a multifunctional 2D perovskite passivation strategy to reduce the photovoltage loss of the PSC with *n*-butylammonium bromide (BABr). This dimensionally graded perovskite formation (DGPF) approach resulted in suppressed nonradiative recombination loss in both the bulk and interface of the perovskite layer. Implementing this strategy, the authors obtained the best-performing device with a maximum PCE of 21.5% and an open-circuit voltage value (*V_OC_*) of 1.24 V in the 1.64 eV perovskite system. S. D. Wolf et al. [22] reported 2D perovskite fragments on the top surface of 3D perovskite in inverted device architecture via the introduction of oleylammonium iodide (OLAI). The resulting best-performing device, based on the 2D/3D perovskite structure, reached an impressive PCE of 24.3% with excellent damp-heat stability. L. Wang et al. [23] formed a 2D PEA_2_PbI_4_ capping layer on top of a 3D Cs_0.05_(MA_0.17_FA_0.83_)_0.95_Pb(I_0.83_Br_0.17_)_3_ perovskite film via post-treatment using phenetylammonium iodide. The 2D layer increased Fermi-level splitting under illumination, improved energy level alignment and reduced non-radiative recombination. As a result, the device efficiencies increased from 17.0% to 18.5% and the long-term stability of the device was also enhanced, maintaining 90% of the initial PCEs after 1000 h under ambient dark shelf storage conditions at 60% relative humidity. X. Zhang et al. [24] applied guanidine (Gu) salts for the dual passivation of 3D perovskite. The bulk of 3D perovskite was doped by GuCl and the surface was treated by GuI. The authors reported a reduction in nonradiative recombination and in the hindering of halogen escape after the treatment. The 2D/3D perovskite solar cells demonstrated enhanced efficiency with PCE values reaching 22.5%, an improvement over the untreated device PCE of 20.3%. Furthermore, the 2D/3D devices exhibited improved stability, retaining over 90% efficiency after storage for 30 days in ambient conditions without encapsulation.

Here, we applied Guanidinium tetrafluoroborate CH_6_BF_4_N_3_ (GATFB) as post-treatment on the hybrid organic–inorganic multi-cation and mixed-halide perovskite film. To the best of our knowledge, this is the first time that GATFB has been used in the post-treatment of the interface of multi-cation and mixed-halide perovskite layer and the hole-transport layer in regularly structured devices. The post-treatment process was carried out by dissolving GATFB in isopropyl alcohol (IPA) to form a solution, which was then spin-coated onto the 3D hybrid perovskite thin film. The effect of the guanidinium salt treatment on the perovskite film morphology and electrical and optical properties, as well as on the device performance, were thoroughly studied via the application of different characterization techniques. The results revealed the multiple positive effects of the GATFB post-treatment. The device efficiencies after treatment increased from 16.8% to 18.5% with the best device efficiency reaching 19.7%.

## 2. Materials and Methods

The device fabrication started with cleaning FTO-coated glass substrates (OPV-Tech, Yingkou, China). The substrate cleaning involved consecutive sonication in detergent, deionized water (DI), acetone, and IPA for 15 min each. After sonication, the substrates were blow-dried by nitrogen gas flow and treated by UV–ozone for 30 min. The electron transport layer (ETL) consisted of two consecutively deposited SnO_2_ quantum dot (QD) and SnO_2_ nanoparticle (NP) layers. The preparation of the SnO_2_ QD precursor solution was performed as described by Z. Ren et al. [25]. The commercial 15% tin (II) oxide dispersion in H_2_O (Alfa Aesar, Haverhill, MA, USA) was diluted with DI water in a [1:5] *v*/*v* ratio to serve as SnO_2_ NP solution. The spin coating of both SnO_2_ QD and NP solutions was performed at 3000 rpm for 30 s. After the spin coating of SnO_2_ QDs, the samples were annealed at 200 °C for 1 h, and after the spin coating of SnO_2_ NPs, the samples were annealed at 150 °C for 30 min.

For the perovskite layer growth, the samples were transferred into the glove box with a controlled inert atmosphere. The perovskite precursor solution was prepared using 1.1 M FAI (Greatcell Solar Materials, Queanbeyan, NSW, Australia), 1.2 M PbI_2_ (99%, Sigma Aldrich, St. Louis, MO, USA), 0.2 M MABr (Greatcell Solar Materials, Queanbeyan, NSW, Australia), 0.4 M MACl (≥99%, Merck, Darmstadt, Germany), and 0.2 M PbBr_2_ (≥98%, Sigma Aldrich, St. Louis, MO, USA) dissolved in 1 mL of a mixture of dimethylformamide (DMF) (Sigma Aldrich) and dimethyl sulfoxide (DMSO) (Sigma Aldrich, St. Louis, MO, USA). The solvent mixture was prepared in advance by mixing DMF and DMSO at a [4:1] *v*/*v* ratio. After dissolving all components, the solution was filtered using 0.45-micron syringe filters. A total of 28 μL of 1.5 M CsI solution in DMSO and 28 μL of 1.5 M RbI solution in [4:1] *v*/*v* mixture of DMF/DMSO were added to the 940 μL of the filtered solution to dope it with Cs and Rb. The spin coating of the perovskite layer was performed via two-step rotation at 1000 rpm for 10 s and at 5000 rpm for 30 s. An amount of 150 μL of chlorobenzene (CB) (Sigma Aldrich, St. Louis, MO, USA) was dropped onto the substrates during the last 10 s of the rotation. The films were further placed onto a hotplate and annealed at 105 °C for 75 min. GATFB (Greatcell Solar Materials, Queanbeyan, NSW, Australia) treatment was performed by dissolving 3 mg of guanidinium salt in 1 mL of IPA and dynamically spin coating the salt solution at 5000 rpm for 30 s on top of formed multi-cation mixed-halide perovskite layer. The samples were further annealed at 100 °C for 5 min. To remove excess material and unreacted components, samples were further washed by IPA and annealed at 100 °C for 5 min.

The hole-transport layer (HTL) was deposited from the solution of 2,2′,7,7′-Tetrakis(N,N-di-p-methoxyphenylamino)-9,9′-spirobifluorene (Spiro-MeOTAD, Lumtec, Taiwan) in CB. The solution was prepared by dissolving 80 mg of Spiro-MeOTAD in 954 μL of CB and adding 29 μL of 4-tert butylpyridine (TBP) (Sigma Aldrich, St. Louis, MO, USA) and 17 μL of lithium salt solution (520 mg of Bis(trifluoromethane)sulfonimide lithium salt (Li-TFSI) (Sigma Aldrich, St. Louis, MO, USA) in 1 mL of acetonitrile (Sigma Aldrich, St. Louis, MO, USA)). The solution was further filtered using 0.45-micron syringe filters and spin coated at 3000 rpm for 30 s inside the nitrogen-filled glove box. For the back electrodes, 70 nm thick gold film was evaporated on top of HTL under the vacuum of 10^−6^ Torr.

Grazing incidence wide angle X-ray scattering (GIWAXS) measurements were performed at the Synchrotron & Printable Electronic Lab, Hoffmann Institute of Advanced Materials, Shenzhen Polytechnic University, with a SAXSFocus 3.0 (GKINST, Hefei, China) equipped with a Cu X-ray source (8.05 keV, 1.54 Å) and a EIGER 2R 500K detector (Bruker, Darmstadt, Germany). The incidence angle was 0.5°. X-ray photoelectron spectroscopy (XPS) and ultraviolet photoelectron spectroscopy (UPS) measurements were performed on an X-ray photoelectron spectrometer NEXSA (Thermo Scientific, Waltham, MA, USA). X-rays were produced by a monochromated low power Al Kα X-ray source at 1486.6 eV. Samples were measured shortly after deposition without additional ion etching. UPS measurements were performed at 10 V bias. Scanning electron microscopy (SEM) images were taken using a Zeiss Crossbeam 540 (Zeiss, Oberkochen, Germany) with a field emission electron source. The electron voltage ranged from 3 to 5 kV. A carbon tape was used to prevent excess charges on the samples by draining them to the sample holder. Atomic force microscopy (AFM) analysis was performed using a Smart SPM 1000 system (Horiba, Palaiseau, France) operating in contact or tapping modes under ambient conditions. Pt-coated cantilevers were used for conductive measurements. The 5 × 5-micron sample areas were studied during each scan. X-ray diffraction analyses were conducted using a Rigaku SmartLab^®^ (Rigaku, Tokyo, Japan) X-ray diffraction (XRD) system. The measurements employed a Cu Kα radiation source at 40 kV and 30 mA. The characterizations were carried out in the 2θ scan mode from 10° to 40° degrees, with an increment of 0.1° degrees. A Perkin Elmer Lambda (Perkin Elmer, Waltham, MA, USA) 1040 UV/Vis/NIR dual beam spectrophotometer equipped with the three-detector (PMT/InGaAs/PbS) module was utilized for absorbance measurements. The wavelength range was selected from 300 nm to 900 nm, with an increment of 2 nm. To capture photoluminescence (PL) and time-resolved photoluminescence (TRPL) signals, the Edinburgh FLSP920 spectrophotometer system (Edinburgh Instruments, Livingston, UK) was employed. The samples were stimulated by a 485 nm picosecond-pulsed diode laser with the power of 0.15 mW. The Oriel EQE 200 (Newport, Irvine, CA, USA) system was applied to perform external quantum efficiency characterizations. A 150 W xenon arc lamp served as a light source and no additional light bias was applied. The chopper frequency was set to 200 Hz. An Agilent B1500A parameter analyzer (Agilent, Santa Clara, CA, USA) was utilized to acquire current–voltage (IV) measurements. The samples were illuminated under 1 sun using light from the Oriel Sol3A solar simulator (Newport, Irvine, CA, USA), equipped with an AM 1.5 G filter (Newport, Irvine, CA, USA). The light intensity was adjusted using a Si reference cell.

## 3. Results and Discussion

**Figure 1:**
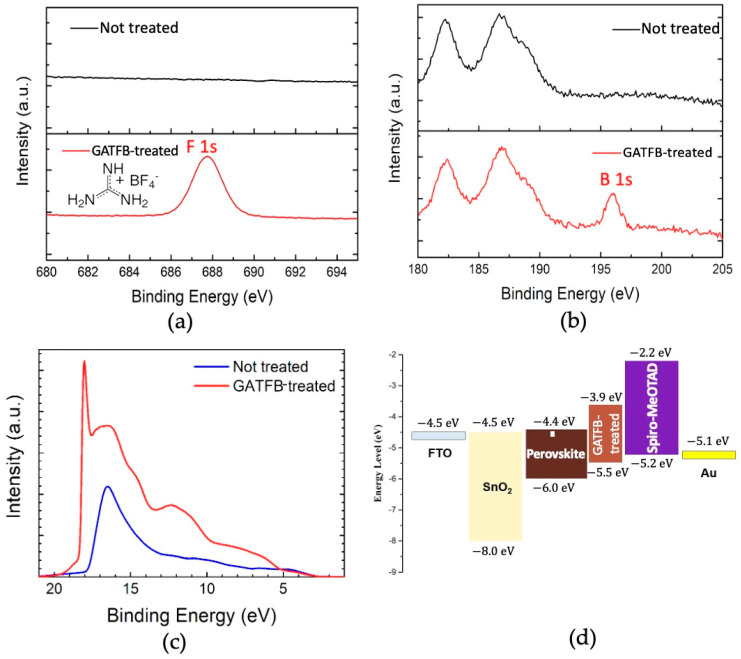
(**a**) XPS spectra of F 1s peak for the control and GATFB-treated perovskite films; the structural formula of GATFB is shown in the inset; (**b**) XPS spectra of B 1s peak for pristine and GATFB-treated perovskite films; (**c**) UPS spectra of untreated and GATFB-treated perovskite films; (**d**) the energy band diagram of the functional layers in a complete device.

The modification of the surface of perovskite films by GATFB treatment were confirmed by X-ray photoelectron spectroscopy (XPS) measurements. The XPS spectra of pristine (control) and GATFB-treated films are shown in Figure 1. Compared with the reference, noticeable signal peaks corresponding to fluorine (F) and boron (B) were found in the films treated with GATFB. The 1s peak of F is shown at 687.7 eV in Figure 1a and 1s peak of B at 196 eV is also present in Figure 1b. These peak energies are consistent with the reported values of F 1s and B 1s in the tetrafluoroborate compound [26,27]. To study the energy band alignment between 3D and 2D perovskite layers, ultraviolet photoelectron spectroscopy (UPS) measurements were performed on untreated and treated films. The UPS spectra of control and GATFB-treated films are shown in Figure 1c. Using the band gap value extracted from absorbance measurements, valence band maxima (VBM) and conduction band minima (CBM) for untreated (3D PVK) and treated films were calculated and represented in the form of energy band diagram in Figure 1d. It can be seen from the figure that GATFB treatment is able to increase the VBM and CBM of the perovskite layer, improving band alignment for carrier extraction and providing an additional barrier to prevent electrons from reaching the hole transporting layer where they can recombine with the holes.

**Figure 2:**
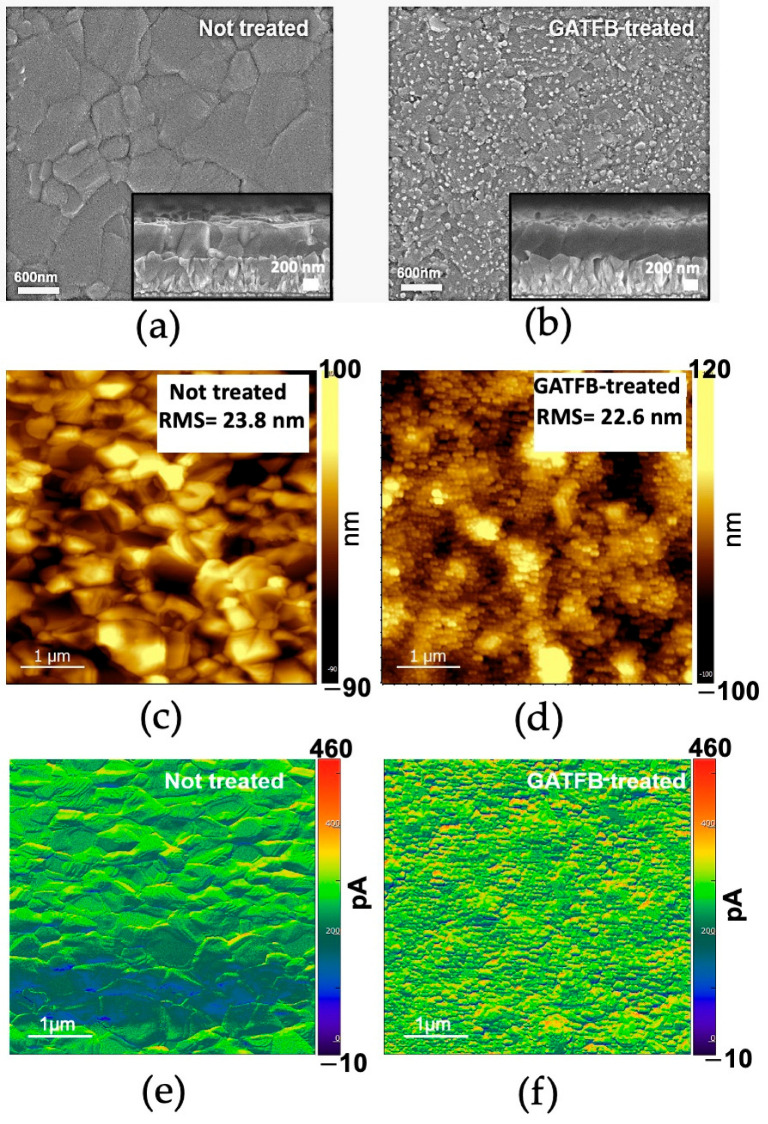
The SEM top-view and cross-section (inset) images of the (**a**) control and (**b**) GATFB-treated perovskite films; AFM topology images of (**c**) control and (**d**) GATFB-treated perovskite films; conductive probe AFM topology images of (**e**) control and (**f**) GATFB-treated perovskite films.

Scanning electron microscopy (SEM) was used to investigate the impact of GATFB post-treatment on the surface morphology of perovskite films. The surface topology SEM images of control and GATFB-treated perovskite films are shown in Figure 2a and Figure 2b, respectively. The figures demonstrate change in surface topology after treatment. The perovskite grain boundaries become less obvious, and some fine structures are noticed after GATFB treatment. Cross-sectional SEM images were acquired of numerous regions within the control and GATFB-treated perovskite thin films to statistically analyze the film thicknesses. The images depicted in the insets of Figure 2a,b show representative cross-sections. The analysis of the images yielded an average thickness of 498 ± 46 nm for the control film and 474 ± 33 nm for the GATFB-treated film, indicating a minor reduction in perovskite layer thickness, resulting from the post-treatment with GATFB dissolved in IPA. The surface topology of control and GATFB-treated perovskite films were further studied using the technique of atomic force microscopy (AFM). AFM images of control and GATFB-treated films are presented in Figure 2c and Figure 2d, respectively. The GATFB-treated perovskite film exhibits a slight reduction in root mean square (RMS) roughness compared to the control sample. A reduction in film roughness is beneficial for growing consecutive layers on top with improved physical contact and reduced interfacial defects [28]. The results obtained from AFM using a conductive probe can study the surface current of the control (Figure 2e) and GATFB-treated (Figure 2f) films. A more homogenous surface current distribution as observed from the GATFB-treated films is indicative of better surface uniformity, which is beneficial for charge transfer processes between the active layer and the consecutive HTL [29].

**Figure 3:**
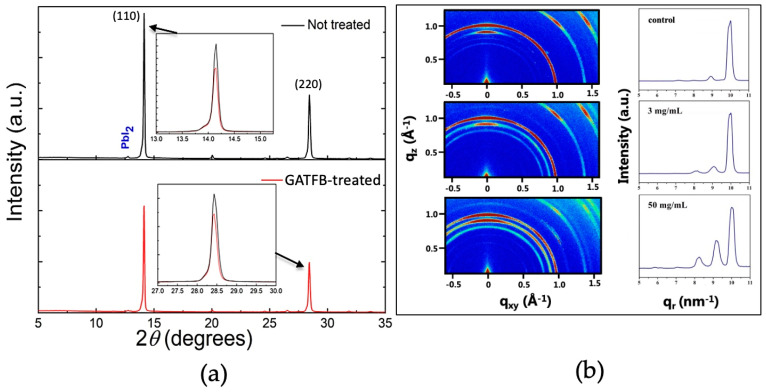
(**a**) XRD spectra of the control and GATFB-treated films. Inserts demonstrate enlarged (110) and (220) plane peaks; (**b**) GIWAXS patterns of perovskite films treated with different concentrations of GATFB.

The phase composition analysis of perovskite films with and without GATFB posttreatment were studied via X-ray diffraction (XRD) measurements. The XRD patterns of the control and GATFB-treated films are presented in Figure 3a. The films with and without GATFB post-treatment both demonstrate characteristic perovskite peaks at 14.1° and 28.3° corresponding to (110) and (220) crystal planes, respectively, indicating that the treatment does not lead to the polymorphism or variation of lattice [8,29,30,31]. A slight shift in the perovskite peaks to lower 2*θ* values suggests the incorporation of larger organic cations into the perovskite lattice, thus enlarging the distances between planes (inserts of Figure 3a). To confirm the formation of the 2D perovskite structures on the surface of 3D bulk perovskite material, grazing incidence wide angle X-ray scattering (GIWAXS) analysis was conducted. The GIWAXS spectra of pristine and GATFB-modified perovskite films are shown in Figure 3b. The pristine sample demonstrates wide Debye–Scherrer rings of the (111) plane at *q* = 10 nm^−1^, indicating isotropic crystallite orientation and signal at *q* = 9 nm^−1^, implying the presence of excess PbI_2_ [32,33]. The GATFB treatment of the 3D perovskite surface creates a new peak at *q* = 8 nm^−1^, which is attributed to the (004) plane of the 2D perovskite [34]. This is evidence of the formation of 2D perovskite on top of 3D perovskite film after the post-treatment [24].

**Figure 4:**
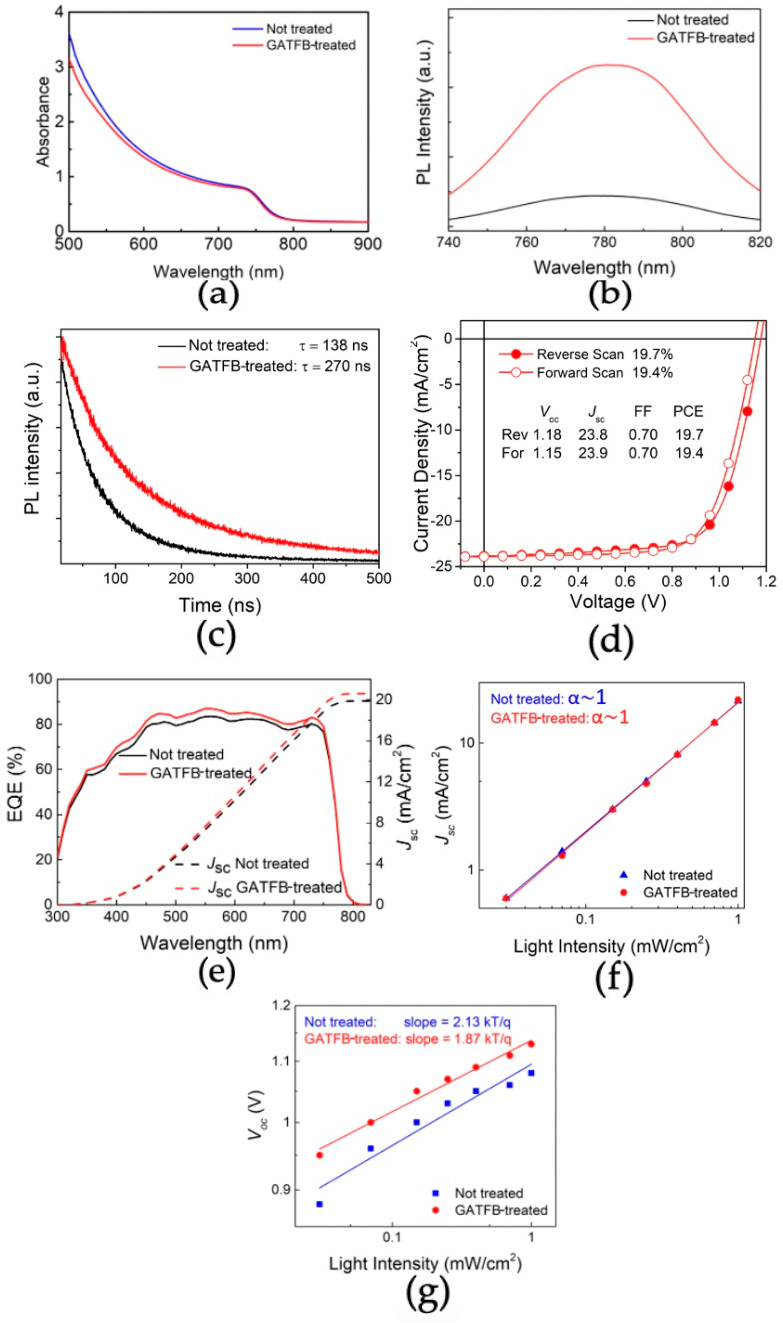
(**a**) Absorbance spectra of the control and GATFB-treated perovskite films; (**b**) PL spectra of the control and GATFB-treated perovskite films; (**c**) TRPL spectra of the control and GATFB-treated perovskite films; (**d**) *JV*-curve of best-performing device and main device parameters; (**e**) EQE and integrated *J_SC_* for the control and GATFB-treated devices; (**f**) *J_SC_* dependence on light intensity in logarithmic scale for untreated and GATFB-treated devices; (**g**) *V_OC_* dependence on light intensity in semi-logarithmic scale for the control and GATFB-treated devices.

The optical characterization of the films was performed via absorbance measurements. The absorbance spectra of the control and GATFB-treated film are presented in Figure 4a. It is consistently noticed that a slight reduction in the absorbance of the perovskite films after GATFB post-treatment. This observation supports the finding of reduced perovskite thickness upon the post-treatment as observed from the SEM cross-section images of the samples. Meanwhile, the band gaps extrapolated from absorbance data yield identical values of 1.61 eV for both the control and GATFB-treated films, indicating that the post-treatment processing used in this work does not significantly impact the intrinsic electronic structure of the perovskite material. The perovskite films were further studied by photoluminescence (PL) spectroscopy. The steady-state PL spectra of the control and GATFB-treated perovskite films deposited on a glass substrate are shown in Figure 4b. The GATFB-treated perovskite film demonstrates an enhancement in the PL signal, which can be attributed to a reduction in non-radiative recombination as a result of the passivation of surface defect states. Furthermore, the time-resolved PL (TRPL) technique was applied to the control and post-treated perovskite films grown on the glass substrate, and the obtained spectra are shown in Figure 4c. The GATFB-treated film exhibits prolonged photoluminescence decay time compared to the control. The TRPL data corroborate results obtained from the steady-state PL measurements, implying a decrease in carrier recombination after the GATFB post-treatment of the perovskite layer.

To study the effect of GATFB post-treatment in a complete device, n-i-p structured PSCs were fabricated, and their performances were characterized. Current–voltage (*IV*) measurements were carried out under AM 1.5 G and 1-sun standard illumination conditions. The extracted average device parameters are summarized in Table 1. It is noteworthy that the GATFB post-treatment leads to an increase in average short-circuit current density (*J_SC_*), raising the value from 22.9 mA/cm^2^ in the control to 23.4 mA/cm^2^ in the post-treated devices. This enhancement in *J_SC_* occurs despite a minor thickness reduction being observed for the post-treated perovskite layers. The rise in photocurrent suggests an improved charge extraction as a result of favorable band alignment in PSCs as previously discussed and a reduction in the carrier recombination as indicated in PL results. The average values of open-circuit voltage (*V_OC_*) and fill factor (*FF*) also increase substantially. The *V_OC_* improvement can be explained by the passivation of shallow trap states resulting in larger quasi-Fermi level splitting [35], whereas *FF* enhancement is attributed to improved charge transport and reduction in carrier recombination. All these contributions result in an increase in the average PCE as well as a reduction in the hysteresis index (HI) for GATFB-treated devices. The highest PCE of the best-performing device reaches 19.7%, and its *JV* curve with the extracted photovoltaic parameters is shown in Figure 4d.

The external quantum efficiency (EQE) measurements were performed without light bias for the control and GATFB-treated devices. The EQE results and *J_SC_* calculated from the EQE data are presented in Figure 4e. The integrated *J_SC_* values are 19.9 mA/cm^2^ and 20.6 mA/cm^2^ for the control and GATFB-treated devices, respectively. The calculated *Jsc* values are ~12–13% lower than corresponding values obtained from the *IV*-measurements. Such discrepancies between the EQE-derived and *J-V J_SC_* are commonly seen in PSCs and can be attributed to the different illumination protocols used. [29]. Nevertheless, the higher *J_SC_* of the post-treated device determined from EQE results supports the trend of the *J-V* results, corroborating the beneficial effect of GATFB post-treatment on the photocurrent of PSCs. The varied light intensity *IV*-measurements were performed to study the charge transfer processes in devices. The *J_SC_* dependence on light intensity in the logarithmic scale for the control and GATFB-treated PSCs are shown in Figure 4f. The plots exhibit a linear dependence of the current density on light intensity with the slope value close to 1 (*J_SC_* = I^α^, α~1) for both cases, implying that the current is not limited by space charges in the device. The *V_OC_* dependence on light intensity in semi-logarithmic scale for the control and GATFB-treated PSCs are plotted in Figure 4g. The slope is represented in terms of *kT/q*, where *k* is the Boltzmann constant, *T* is the absolute temperature, and *q* is the elementary charge. The GATFB post-treated device demonstrates a reduction in slope compared to the control device: 1.87 *kT/q* < 2.13 *kT/q*. This implies a reduction in trap-assisted recombination in GATFB-treated devices, supporting previous findings from PL and *IV*-measurements.

## 4. Conclusions

This work applied GATFB as a post-treatment to modify the surface of multi-cation mixed-halide perovskite. A series of characterizations were performed to study the effects of the GATFB post-treatment on perovskite films as well as solar cell performance. The experimental results show that low-dimensional perovskite structures are formed atop the 3D perovskite after post-treatment. The photocurrents of PSCs are increased despite a slight reduction in the perovskite thickness after GATFB post-treatment. The post-treated PSCs demonstrate a PCE of 18.5% on average, with a highest efficiency of 19.7% and a *V_OC_* of 1.18 V. The enhancement of the photovoltaic performance is attributed to the improved energy band alignment in PSCs. The presence of low-dimensional perovskite structures at the interface of PSCs also suppresses non-radiative recombination and enhances the carrier extraction of devices. The multiple positive effects of the GATFB post-treatment on PSCs have been demonstrated experimentally and suggest the potential of using GATFB for interface engineering to further optimize PSCs.

## Figures and Tables

**Table 1:** Device parameters for untreated and GATFB-treated perovskite solar cells. Averages were obtained from seven devices.

Type	Scan	*V_OC_* (V)	*J_SC_* (mA/cm^2^)	*FF*	PCE (%)	HI
Not treated	RS	1.12 ± 0.02	22.9 ± 0.4	0.66 ± 0.02	16.8 ± 0.9	0.07 ± 0.03
	FS	1.13 ± 0.02	22.9 ± 0.9	0.70 ± 0.01	18.0 ± 0.6	
GATFB-treated	RS	1.16 ± 0.01	23.4 ± 0.4	0.68 ± 0.03	18.5 ± 0.9	0.02 ± 0.02
	FS	1.14 ± 0.01	23.4 ± 0.4	0.69 ± 0.01	18.5 ± 0.5	

## Data Availability

Dataset available upon request from the authors.
